# Knowledge, attitude, and practices related to COVID-19 among poor and marginalized communities in central India: A cross-sectional study

**DOI:** 10.1371/journal.pone.0264639

**Published:** 2022-04-06

**Authors:** Krithika Murali, Nitya Balagopalan, Jyoti Benawri, Anand Kumar Bairagi, Nagappa Veerappa Heggannanavar, Ashish Srivastava, Swati Mahajan

**Affiliations:** 1 Jhpiego, New Delhi, India; 2 Jhpiego, Madhya Pradesh, India; 3 Jhpiego, Jharkhand, India; Universiti Teknologi Malaysia - Main Campus Skudai: Universiti Teknologi Malaysia, MALAYSIA

## Abstract

COVID-19 has led to unprecedented challenges and requires local and global efforts for its mitigation. Poor and marginalized populations are more vulnerable to the health, social and economic effects of the pandemic. The objective of this study was to know about the knowledge, attitude and practices towards COVID-19 among poor and marginalized communities in central India and the factors associated with them so that effective risk communication messages can be designed and community engagement needs and strategies can be identified. A cross-sectional survey was conducted using an Interactive Voice Response System as part of the NISHTHA-Swasthya Vani intervention, which is a platform for dissemination of key messages related to COVID-19, social welfare schemes, national health programs and other important information. A total of 1673 respondents participated in the survey. The mean knowledge, attitude and practice scores of the respondents was 4.06 (SD = 1.67) out of 8, 2.46 (SD = 1.18) out of 4 and 3.65 (SD = 0.73) out of 4 respectively. More than 50% respondents exhibited stigma towards recovered COVID-19 patients(n = 347) and towards health workers(n = 384) catering to COVID-19 patients. The factors associated with higher KAP scores were education, occupation, age and primary source of information on COVID-19. There was a positive correlation between knowledge and attitude (co-efficient: 0.32) and a negative correlation between knowledge and stigma (co-efficient: -0.28). The knowledge, and attitude scores related to COVID-19 were low among the poor and marginalized communities, while the prevalence of stigma was high. Therefore, there is a need for effective risk communication for these communities through alternate channels.

## Introduction

COVID-19 caused by SARS-COV-2 virus is one of the most significant pandemics the world has seen in recent decades [1]. It has led to unprecedented challenges in all sectors- health, social and economic and has called for coordinated efforts globally and locally for its mitigation [2]. We are currently in the second year of the pandemic leading to millions of cases and deaths worldwide [3].

Low and middle-income countries (LMICs), have borne the brunt of the pandemic in comparison to other developed nations given their already overburdened health systems prior to the pandemic and unique challenges of limited resources and poor infrastructure, lack of access to reliable information for households in resource-poor settings and competing health priorities [4, 5]. In addition, LMICs face more challenges in rolling out non-pharmaceutical interventions like lockdowns, as their associated outcomes such as loss of employment, income, or disruption of food or water would affect people’s ability to adhere to lockdown conditions and also their health and wellbeing [6].

In India, the first case was detected on 27^th^ January, 2020 [7]. During the first wave a prompt nationwide lockdown was imposed in March, 2020, which helped in containing the pandemic. The subsequent easing of restrictions and resumption of economic activities in an uncoordinated manner resulted in a second wave of infections that stretched the country’s health system beyond capacity and saw a large number of deaths [8]. This clearly highlights that a country like India, which is the second most populous country in the world, is densely populated and has a high internally mobile population [9], will always be at risk of multiple waves of infections and adverse impact, till the pandemic is over.

Earlier outbreaks like SARS and Ebola have highlighted the importance of risk communication and community engagement for health emergency readiness and response activities [10]. Timely and proactive communication on what is known, what is unknown, and what is being done to get more information, plays an important role in gaining the trust of the communities [11].

Experiences from past infectious disease outbreaks(e.g H1N1, Bubonic plague, HIV, Tuberculosis, cholera, Zika virus, SARS) have shown that they were accompanied with misinformation, myths, associated stigma etc., which hindered the containment and mitigation measures taken by the authorities [12]. During the Ebola outbreak in Nigeria and Guinea, the misconceptions regarding the disease led to lack of preventative behaviour as well as mistrust and attack on health care providers [13].

Similarly, for COVID-19, there is a strong need to adopt a locale-specific approach to create awareness among specific clusters of population regarding prevention, spread and treatment of COVID-19 [14]. The World Health Organization (WHO) lists risk communication and community engagement as an important measure to flatten the transmission curve and mitigate the impact of the COVID-19 pandemic [11]. This can be done only if there is an understanding of the community’s readiness to adopt behaviour practices and measures suggested by health authorities [15]. Assessment of the knowledge, attitude and practices (KAP) of people towards COVID-19 will play an integral role in understanding and adopting specific approaches for controlling of spread of COVID-19 [16].

A particularly vulnerable group of population are those belonging to the lower socio-economic strata, depending on daily wages for survival and having limited access to internet, media etc [17, 18]. These populations are at greater risk of getting infected, which is amplified by their lack of information and access to quality healthcare as well as their already compromised socio-economic determinants [19].

### Program

The United States Agency for International Development (USAID)’s flagship health system strengthening project NISHTHA is working across 13 states in India and providing technical support to strengthen the government’s response for COVID-19. NISHTHA is a Hindi word meaning commitment signifying the project’s commitment towards strengthening primary health care. To support the above described vulnerable communities during COVID-19 and to ensure they have access to timely and correct information, NISHTHA set up a communication platform to reach the target population through an alternate medium and link them for appropriate care through a network of local partners. The risk communication platform-NISHTHA-Swasthya Vaani was rolled out in the month of August, 2020 in four districts of Madhya Pradesh state (Guna, Rajgarh, Khandwa and Barwani) and one district of Jharkhand (Ranchi) state.

The communication platform used an Integrated Voice Response system (IVRS). It was developed by Gram Vaani- a social tech company–as an innovative social media platform for the non-internet users living in rural areas. This platform was chosen for risk communication as the smart phone and internet penetration in these regions, particularly in the rural areas is very low [20]. However, the mobile phone penetration is considerably higher with over three-fourths of the population using mobile phones [20].

A toll-free number was generated where community members could access information regarding COVID-19 through the IVRS. Information about the toll-free number and the NISHTHA-Swasthya Vani platform was disseminated to the community through pamphlet distribution, IEC material display at prominent locations (health centres, shops etc.) in the community and through active reach out by community volunteers and frontline workers, particularly in rural areas.

This platform provided risk communication messages to the vulnerable populations including migrant workers and their families, elderly, persons with disabilities, urban poor and other people living in remote areas. The messages related to COVID-appropriate behaviours, basic information on COVID-19, anti-stigma, anti-discrimination and countering misinformation.

As an additional part of the intervention, in order to further tailor the messages to the targeted population served by the project, a survey was administered to understand the existing knowledge attitude and practices towards COVID-19, so that effective risk communication messages could be designed for addressing the knowledge, attitude and practice gaps.

The primary objective of this survey was to know the knowledge, attitudes and practices of poor and marginalized communities towards COVID-19 and determine the factors associated with it.

## Methodology

### Study design

This was a cross-sectional study done using a structured questionnaire, administered through an IVR system.

The survey was conducted using the same IVRS platform that was used to disseminate risk communication and awareness messages. Due to limitations of the IVRS platform, a limited number of questions were incorporated into the survey instrument.

### Study site

This study was part of the NISHTHA-Swasthya Vaani Program which was implemented in 4 districts of Madhya Pradesh and 1 district of Jharkhand. Four out of these five districts are aspirational districts and have ongoing NISHTHA interventions. Aspirational districts are those identified districts by Government of India that have shown relatively lesser progress in key social areas, and have emerged as pockets of under-development [21]. In addition, rural areas of these five districts have a very high incidence of poverty (range from 41.65% to 70.72%) when compared with the national incidence (25.01%) [22].

### Study population

The study population included all the people living in the Ranchi district of Jharkhand and Khandwa, Guna, Barwani and Rajgarh district of Madhya Pradesh. Ranchi is the capital city of Jharkhand with a population of more than 2.9 million, which is 8.8% of the population on Jharkhand and has a literacy rate of 76% [23]. Khandwa, Rajgarh, Guna and Barwani have a population of 0.2 million, 1.5 million, 1.2 million and 1.3 million respectively and literacy rates of 66%, 61% 63% and 49% respectively [24].

### Study participants

People from all the five districts were informed about the platform by displaying the toll-free number in prominent areas such as health facilities, chemists, shops etc. In addition, a set of community volunteers as well as frontline health workers informed the people belonging to the above described vulnerable group, about the platform and IVRS number through outreach activities in the rural areas. People who were interested in listening to the messages called the IVRS number. All the first-time callers who accessed the NISHTHA- Swasthya Vaani platform by dialling-in the toll-free number in the first 3-months since its launch were directed to the survey. Those who opted to participate, and answered 4 or more questions were included in the study.

### Study duration

This study was conducted in a time period of 3 months from August-2020 to October 2020.

### Study instrument

The study tool was a structured questionnaire, developed on the basis of literature review [25–29], existing guidelines given by WHO, Government of India and Indian Council of Medical Research(ICMR) and with inputs from field experts who have professional degrees in medicine and public health, and have experience of working with the target population. The questionnaire was also shared with government program managers of the states and respective districts for feedback before opening the survey for the community on the IVRS platform. It was also reviewed by team Gram Vaani, who are experts in utilizing IVR based platforms for conducting community surveys [30], to check for its appropriateness for the IVR platform.

The survey was administered in two parts- a short questionnaire and a long questionnaire so that the loss of respondents over the course of the survey could be minimized. The short questionnaire consisted of questions on socio-demographic details such as age, gender, district, marital status, occupation and education, source of information and five questions assessing knowledge and attitudes. The long-questionnaire consisted of nine questions further assessing the knowledge, attitudes and practices.

The questions pertaining to knowledge asked about common symptoms, mode of spread, prevention, steps to take and people at increased risk. Questions pertaining to attitude asked about concern regarding COVID-19, attitude towards social gathering, as well as questions related to stigma towards patients and healthcare workers. The practices section had questions regarding mask wearing and hand washing **(S1 Appendix)**.

### Study procedure

When the users dialled in the toll-free number, they were first read out an automated introductory message and instructions for the survey. Everyone who accessed the platform, was asked to answer questions on socio-demographic characteristics, after which they were directed to the short questionnaire-which assessed the knowledge and attitude towards COVID-19. The questions and answer options were read out in the local language-Hindi. The respondents could choose their answer by dialling in the option number. The questions were not mandatory and could be skipped. In addition, the users had the option of listening to the question again by dialling in a number. Post completion of the short questionnaire, the users were given the option of proceeding to the long questionnaire or accessing the available content on platform. Those who proceeded to the long survey were asked additional questions on knowledge, attitude and practices towards COVID-19. The respondents could not go back to the survey after accessing the content and on repeat calling, to avoid duplication and bias.

### Statistical analysis

The data were extracted from the IVR platform in comma separated values (CSV) format and exported to SPSS Statistics for Windows, version 17.0 (SPSS Inc., Chicago, Ill., USA) for further analysis. The socio-demographic characteristics and survey responses were recorded as frequencies and percentages. The mean scores for the domains of knowledge, attitude and practices was calculated after scoring the individual responses. Each knowledge response was scored as 1 for correct answer and 0 for incorrect answer. The attitude responses were scores as 1 for desirable and 0 for undesirable. The practice responses were scored as 0 for never, 1 for occasionally and 2 for always. The scoring for the domains ranged as follows: 0–8 for knowledge, 0–4 for attitude and 0–4 for practices. Additionally, 2 of the attitude questions constituted the stigma score ranging from 0–2. We converted mean scores to percentages and used a cut-off of 80% for categorizing low and high scores as per Blooms cut-off [31]. The mean and standard deviation of the scores was calculated. The mean scores were compared based on socio-demographic parameters using ANOVA F-test. Although the distribution was non-normal for some of the categories, due to the fairly large sample size of the study, in accordance with the central limit theorem and the robustness of the ANOVA test; we decided to consider the ANOVA results and opted for the same [32, 33]. While comparing the means using ANOVA, the homogeneity of variances was tested using Levene’s test for homogeneity and wherever the assumption of homogeneity of variance was violated, we have presented the Welch ANOVA results. The post hoc tests were applied wherever ANOVA/Welch ANOVA was significant using Tukey HSD test for ANOVA and Games Howell test for Welch ANOVA. Correlation between knowledge, attitude and practices score was assessed using Pearsons Correlation. Correlation of stigma score with knowledge was also done. A correlation coefficient of upto 0.3 was regarded as weak correlation, from 0.3 to 0.7 was regarded as moderate correlation and greater than 0.7 regarded as strong correlation [34]. Multivariable linear regression was run using enter method for the knowledge and attitude scores with independent variables: age, gender, marital status, education, occupation and main source of information. The models were run after testing for assumptions of linearity, normality, homoscedasticity and independence of observations. In addition, they were also assessed for multi-collinearity and presence of outliers. For the linear regression model on attitude, the knowledge score was also included as an independent variable. Regression was not run for practice scores, as the assumptions of linearity and normality were not met. The significance level for all the tests was set at P = 0.05.

### Ethical considerations

The Institutional Review Board (IRB) of the Johns Hopkins Bloomberg School of Public Health, USA determined the study as a public health surveillance activity (IRB No:15306). Requisite permissions for rolling out the platform in five districts were also obtained from the state governments of Madhya Pradesh and Jharkhand.

Considering the survey was administered using an IVRS platform, it was not possible to obtain consent from individual participants. However, in accordance with the determination notice, the participants were notified about the survey through an oral script that was played at the start of the survey explaining the purpose and procedure of the survey. The participation in the survey was voluntary and all community members could access the information even if they did not wish to take part in the survey.

In India, the legal age for acquiring a mobile phone number in India is 18 years, and because the survey required voluntary participation by dialling-in the IVR number, it was considered that all participants below 18 yrs of age, had parental or guardian consent for accessing a mobile phone and participation in survey.

## Results

### Socio—Demographic characteristics

Across 5 districts, a total of 24,483 calls were either made to or received from the NISHTHA-Swasthya Vaani channel in the period between August to October, 2020. These included 6672 first-time callers to the toll-free number, who were directed to the short survey. Of these, 3396 individuals answered at least 1 question of the short survey and 1289 completed it, who were then directed to the long survey. Of these, 861 participated in the long survey and 577 completed it. The mean duration of time spent by respondents on each of the two surveys was around 3 minutes.

**Fig 1:**
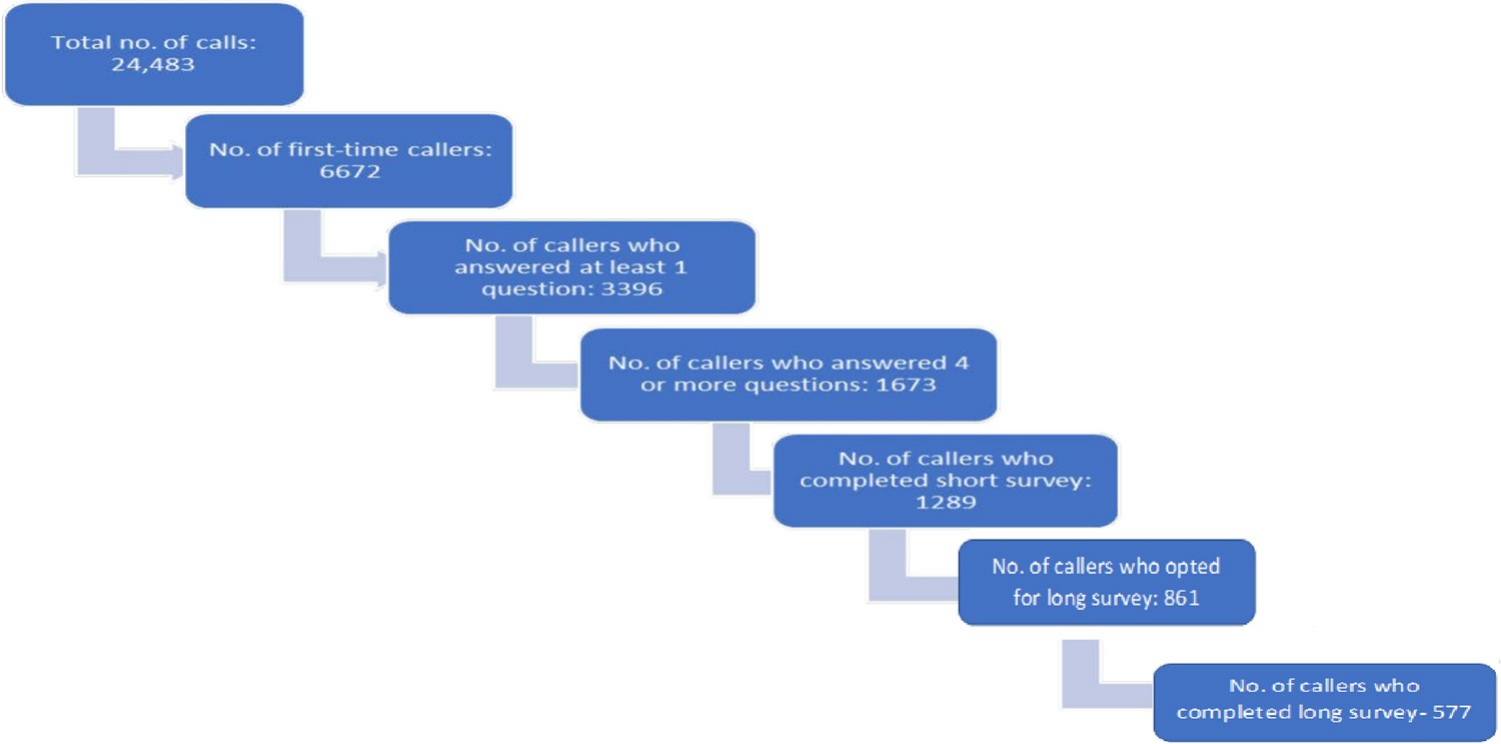
Status of respondents in survey.

For ensuring data quality as well as to minimize the number of no responses, only those 1673 individuals who answered at least 4 out of 14 KAP questions were included in the analysis. The status of users of the platform and participants in each survey, between August to October, 2020 is shown in **Fig 1**.

Among the respondents included in the analysis, 66.7% belonged to the age group of 16–35 years and 63.4% were male. Out of all the respondents, 42.6% had an education from standard 6th to 12th while 17.1% of them had no formal education. Agriculture or daily wage labourers represented 32.1%respondents, followed by Farmers (26%). Unmarried individuals constituted majority (55.5%) of the survey respondents. Detailed demographic characteristics are shown in **Table 1**.

**Table 1:** Socio-demographic characteristics of the survey respondents (n = 1673).

Variable	Characteristics	No. of respondents	Total percent	Valid percent*
**District**	Barwani	78	4.7%	6.6%
	Guna	183	10.9%	10.6%
	Khandwa	232	13.9%	23.6%
	Rajagarh	176	10.5%	10.3%
	Ranchi	1004	60.0%	48.9%
	**Total**	**1673**	**100.0%**	100.0%
**Age**	< 16 years	175	10.5%	13.1%
	16 to 35 Years	1066	63.7%	66.7%
	36 to 55 Years	292	17.5%	17.6%
	>55 years	32	1.9%	2.7%
	No response	108	6.5%	
	**Total**	**1673**	**100.0%**	100.0%
**Gender**	Male	1003	60.0%	63.4%
	Female	531	31.7%	32.3%
	Others	53	3.2%	4.2%
	No response	86	5.1%	
	**Total**	**1673**	**100.0%**	100.0%
**Occupation**	Agriculture or daily wage labour	465	27.8%	32.1%
	Farmer	419	25.0%	26.0%
	housewife or house husband	67	4.0%	4.8%
	salaried employee	246	14.7%	14.4%
	Student	189	11.3%	9.4%
	Self employed	140	8.4%	5.0%
	Unemployed	84	5.0%	8.3%
	No response	63	3.8%	
	**Total**	**1673**	**100.0%**	100.0%
**Marital status**	Unmarried	919	54.9%	55.5%
	Married	658	39.3%	40.7%
	Divorced/Widowed	57	3.4%	3.8%
	No response	39	2.3%	
	**Total**	**1673**	**100.0%**	100.0%
**Education**	No formal education	254	15.2%	17.1%
	Up to grade 5	363	21.7%	22.4%
	Grade 6–12	709	42.4%	42.6%
	Diploma Graduation Post graduation	301	18.0%	17.9%
	No response	46	2.7%	
	**Total**	**1673**	**100.0%**	100.0%

* Valid Percent is the percentage of the categories after excluding the non-responses.

### Source of information

More than half of the respondents (59.5%) reported Radio, TV or newspaper as their main source of information on COVID-19. About 14% respondents reported that the local health workers or the local health facilities were their main source of information on COVID-19.

### Assessment of knowledge

Only around one-third (35.3%) of participants were aware that fever and dry cough are symptoms of COVID-19 infection, and more than three-fourth of the participants knew that people with chronic diseases such as diabetes, heart disease, obesity are at higher risk (77.8%). When asked about steps one can take to prevent infection, less than half (40.3%) selected the correct option of washing hands with soap and water frequently. More than a quarter of the respondents (34.1%) believed that people with COVID-19 infection, who do not have any symptoms, cannot spread infection to others.

A large (59%) proportion of the participants were not aware about minimum physical distance of 2 meters to protect oneself from infection, almost 53% respondents either thought that children and teenagers do not need to make efforts to prevent infection because they have a strong immune system or were not sure. The responses for knowledge scores are as depicted in **Table 2**. The mean knowledge score for participants was 4.06 (SD = 1.67, range 0–8).

**Table 2:** Responses to questions assessing knowledge of survey participants regarding COVID-19.

Question Responses	Frequency	Total Percentage	Valid percentage**
**Q1. Which of the following are common symptoms of CORONA infection? #**			
Fever and dry cough*	562	33.6%	35.3%
Loss of appetite and loss of weight	297	17.8%	18.6%
Itching of skin and rashes	222	13.3%	13.9%
Do not know	514	30.7%	32.2%
No response	78	4.7%	
**Total**	**1673**	**100.0%**	100.0%
**Q2. By which of the following ways does CORONA infection spread? #**			
Respiratory droplets from infected person*	981	58.6%	59.9%
Drinking contaminated water	222	13.3%	13.6%
Mosquito bites	100	6.0%	6.1%
Do not know	333	19.9%	20.4%
No response	37	2.2%	
**Total**	**1673**	**100.0%**	100.0%
**Q3. Which of the following steps can one take to prevent CORONA infection? #**			
Washing hands with soap and water frequently*	671	40.1%	40.3%
Drinking warm water frequently	423	25.3%	25.5%
Standing in sun daily for at least 30 mins	78	4.7%	4.6%
All of the above	418	25.0%	25.2%
Do not know	71	4.2%	4.4%
No response	12	0.7%	
**Total**	**1673**	**100.0**	100.0%
**Q.4 What should one do if someone has symptoms of CORONA infection? #**			
call the CORONA helpline and follow instructions*	782	46.7%	47.4%
take home remedies and avoid going out	146	8.7%	8.8%
Visit the nearest corona hospital	209	12.5%	12.7%
Visit the nearest health facility	415	24.8%	25.2%
Do not know	98	5.9%	5.9%
No response	23	1.4%	
**Total**	**1673**	**100.0%**	100.0%
**Q.5 A minimum distance of how many meters from another person is necessary for protection against CORONA infection? $**			
1 meter	368	42.8%	43.2%
2 meters*^μ^	352	41%	41.3%
3 meters	102	11.8%	12.0%
Do not know	30	3.4%	3.5%
No response	9	1.0%	
**Total**	861	100.0%	100.0%
**Q.6 People with CORONA infection who do not have any symptoms cannot spread infection to others. $**			
True	268	31.1%	34.1%
False*	368	42.7%	46.1%
Not sure	149	17.3%	19%
No response	76	8.9%	
**Total**	861	**100%**	100%
**Q7. Children and teenagers do not need to make efforts to prevent CORONA infection because they have a strong immune system. $**			
True	301	35.0%	42.1%
False*	338	39.2%	47.3%
Not sure	76	8.9%	10.6%
No response	146	16.9%	
**Total**	861	**100%**	100%
**Q8. People with CORONA infection who have chronic diseases such as diabetes, heart disease, and obesity are at higher risk. $**			
True*	549	63.8%	77.8%
False	109	12.6%	15.1%
Not sure	66	7.6%	9.1%
No response	137	16%	
**Total**	861	**100%**	100%

Note: # - short survey questions

$ - long survey questions

*- correct response

μ-as per Government of India guidelines.

** Valid percentage means the percentage of the categories after excluding the non responses.

### Assessment of attitude

Risk perception, prevention intentions and thoughts regarding COVID-19 infected persons and care takers were analysed. Almost one-third (32.9%) of participants reported that they constantly worried about contracting COVID-19 infection. When asked whether there is a chance of the infection spreading if a crowd happens for a religious purpose, 30.7% were either not sure or thought there was no risk of spread in a religious gathering. More than one- third (37.9%) of the participants agreed that for the safety of people, patients who have recovered from COVID-19 should not be allowed to live in the area while 9.7% were not sure. Further, 34.1% of the participants agreed that for the safety of people, healthcare workers taking care of COVID-19 patients, should not be allowed to visit their area while 5.8% were not sure. The responses of participants for questions on attitude are as depicted in **Table 3.** The mean attitude score of the participants was 2.46 (SD = 1.18, range 0–4)

**Table 3:** Responses to questions assessing attitude of survey participants towards COVID 19.

Question responses	Frequency	Total Percentage	Valid percentage **
**Q.1 Do you worry about contracting CORONA infection? #**			
No	460	27.5%	30.2%
Yes, Sometimes	334	20.0%	21.2%
Yes, Many times	238	14.2%	15.7%
Yes, at All times	502	30.0%	32.9%
No response	139	8.3%	
Total	1673	100.0%	100%
**Q.2 If a crowd happens due to religious purpose, there is no chance of CORONA infection spreading $**			
Agree	149	17.4%	21.4%
Disagree	483	56%	69.3%
Not sure	65	7.6%	9.3%
No response	164	19%	
Total	861	100%	100%
**Q.3 For the safety of people in your area, patients who have recovered from Corona infection should not be allowed to live in your area. $**			
Agree	251	29.2%	37.9%
Disagree	347	40.3%	52.4%
Not sure	64	7.4%	9.7%
No response	199	23.1%	
Total	861	100%	100%
**Q.4 For the safety of people in your area, healthcare workers taking care of CORONA patients, should not be allowed to visit your area $**			
Agree	218	25.3%	34.1%
Disagree	384	44.6%	60.1%
Not sure	37	4.3%	5.8%
No response	222	25.8%	
Total	861	100	100%

Note: # - short survey questions

$ - long survey questions.

**- Valid percentage means the percentage of the categories after excluding the non responses.

### Assessment of practice

More than 80% respondents reported that they always wear a mask whenever they were outside and washed hands with soap and water frequently whereas only 15% reported not following the preventative measures at all times. The practices as reported by the respondents is as shown in **Table 4.** The mean practice scores were reported to be 3.65 (SD = 0.73, range 0–4).

**Table 4:** Responses to questions assessing practices of survey participants related to COVID 19.

Question responses	Frequency	Total Percentage	Valid Percentage **
**Q.1 In the past few days, have you worn a mask when you were outside? $**			
Never	21	2.4%	3%
Occasionally	95	11%	13.8%
Always	573	66.6%	83.2%
No response	172	20%	
**Total**	861	**100%**	**100%**
**Q.2 In the past few days, have you been washing your hands with soap and water frequently? $**			
Never	12	1.4%	1.7%
Occasionally	77	9.1%	11.2%
Always	599	69.5%	87.1%
No response	173	20%	
**Total**	12	**100%**	**100%**

Note:# - short survey questions

$ - long survey questions.

** Valid percentage means the percentage of the categories after excluding the non responses.

Mean knowledge score differed significantly across district, education, occupation, main source of information and education level as depicted in **Table 5**. The knowledge scores were significantly higher for those who stated that their main source of information was radio, TV or newspaper as compared to no source. The knowledge, attitude and practice scores were lower for farmers, agriculture and daily wage labours as compared to salaried employees, although there was no significant difference in practice scores across occupation groups.

**Table 5:** Mean knowledge, attitude and practices score by socio demographic parameters.

Variable	Knowledge score (out of 8)		Attitude score (out of 4)		Practices score(out of 4)	
	N	Mean± S.D	N	Mean± S.D	N	Mean± S.D
**Districts**						
**Barmani**	27	4.13±1.65	31	2.7 ±1.2	34	3.85±0.436*
**Khandwa**	57	3.65±1.63	49	2.48±1.13	59	3.75± 0.575
**Rajagarh**	64	3.66±1.5	66	2.4±1.06	70	3.53±0.737*
**Guna**	54	3.78±1.7	54	2.5±1.2	56	3.50±0.831*
**Ranchi**	422	4.22±1.7	420	2.44±1.19	465	3.70±0.636*
**Total**	624	4.07 ±1.7	620	2.46±1.18	684	3.68 ± 0.655
**P-value**	**0.01** ^**#**^		**0.66**		**<0.01** ^**€**^	
**Main Source of COVID-19 information**						
**Friends and relatives**	54	3.69 ±1.58	52	2.5±1.17	60	3.43±0.90***
**Internet**	77	3.99±1.75	74	2.51±1.08	83	3.6±0.79
**Local health worker or local health facility**	60	3.93±1.48	82	2.50±1.08	82	3.82±0.46***
**Radio, TV or newspaper**	402	4.23 ±1.67**	418	2.48±1.20	418	3.67 ±0.73
**No source**	29	3.07 ±1.48 **	32	2.12± 1.15	35	3.43±0.81
**Total**	622	4.08±1.7	614	2.50±1.17	678	3.65±0.73
**P-value**	**<0.01**		0.55		**<0.01** ^**€**^	
**Age groups**						
**Less than 16**	93	3.62±1.61^μ^	88	2.14±1.07 ^μ^	93	3.38±0.95 ^μ^
**16–35**	415	4.13±1.7^μ^	408	2.52±1.17 ^μ^	447	3.67±0.70 ^μ^
**36–55**	86	4.31±1.8^μ^	87	2.71±1.22	98	3.76±0.57 ^μ^
**More than 55**	9	4.56±1.31	9	2.77±1.20	11	3.91±0.302
**Total**	603	4.08±1.7	592	2.50±1.17	649	3.65±0.73
**P-value**	0.02		<0.01		**<0.01** ^**€**^	
**Gender**						
**Male**	411	4.13 ±1.7	406	2.45±1.17	439	3.73±0.70
**Female**	181	4.05 ±1.7	181	2.56±1.17	202	3.61±0.75
**Others**	16	3.38±1.4	13	2.38±1.19	18	3.61±0.778
**Total**	608	4.09 ±1.7	600	2.48±1.18	659	3.65±0.73
**P-value**	0.19		0.57		0.13^€^	
**Occupation**						
**Agriculture or Daily wage labour**	114	4.11±1.83^##^	103	2.48±1.19	119	3.47±0.92^####^
**Farmer**	156	3.85±1.61	154	2.29±1.13^###^	166	3.59±0.83^####^
**Housewife or House husband**	29	4.00±1.75	28	2.03±1.17^###^	30	3.67±0.711
**Salaried employee**	83	4.14 ±1.50^##^	84	2.85±1.15^###^	100	3.78±0.504^####^
**Student**	135	4.21±1.70	136	2.43±1.22	140	3.65±0.748
**Unemployed**	47	4.51±1.6	49	2.63±0.96	53	3.77±0.50
**Self-employed**	48	3.92±1.60	48	2.56±1.23	56	3.80±0.483
**Total**	612	4.02±1.7	602	2.48±1.18	664	3.65±0.73
**P-value**	**0.02**		**<0.01**		**0.02** ^**€**^	
**Marital status**						
**Unmarried**	284	3.99±1.68	286	2.52±1.16	329	3.70±0.68
**Married**	316	4.14 ±1.66	308	2.42±1.17	325	3.61±0.77
**divorced/widowed**	14	4.21±1.71	13	2.76±1.01	17	3.29±1.04
**Total**	614	4.07 ±1.67	607	2.48±1.17	671	3.65±0.74
**P-value**	**0.53**		**0.41**		**0.10** ^**€**^	
**Education**						
**No formal education**	97	3.66±1.58	92	2.19±1.21	105	3.54±0.84
**Up to 5**	110	4.13 ±1.79	111	2.15±1.19	123	3.58±0.82
**6–12**	277	4.11 ±1.74	272	2.50±1.16	298	3.61±0.77
**Diploma Graduation Postgraduation**	129	4.31±1.44 ^ß^	133	2.93±1.01^ß^	143	3.83±0.444 ^ß^
**Total**	613	4.08±1.7	608	2.48±1.18	669	3.65±0.74
**P-value**	**0.02** ^**€**^		**<0.01** ^**€**^		**<0.01** ^**€**^	

Note

^€:^ Homogeneity assumption violated, p-value represents for Welch ANOVA.

^#^: There was no significant difference found on post-hoc analysis.

*: The practice scores of Barwani was significantly greater than Guna, Rajagarh and Ranchi.

**: The knowledge scores was significantly higher for those who said the main source of information radio, TV, newspaper than those who claimed they did not have a source.

***The practice scores were higher for those who said their main source of information was local health worker/facility than those with friends or relatives as main source of information.

μ: The knowledge, attitude and practice scores were significantly lower for less than 16 than rest of age groups.

## the knowledge scores were significantly higher in salaried employees than farmers.

###: The attitude scores were significantly higher in salaried employees than housewives/househusbands and farmers.

####: The practice scores were significantly higher in salaried employees than farmers and agricultural or daily wage labours.

ß: The knowledge, attitude and practice scores were higher in those with higher education than all of the groups.

The knowledge, attitude and practice scores were significantly higher for those in the age group between 36–55 and 16 to 35 than younger respondents. The knowledge, attitude and practice scores were significantly higher for those who had received higher education, as compared to those who had lesser school education. There was no significant difference between the knowledge, attitude and practice scores of males and females.

#### Correlation between knowledge, attitude and practices

Assessment of correlation demonstrated that knowledge has a moderate positive correlation with attitude and a weak positive correlation with practices, whereas attitude and practices were also weakly correlated as shown in **Table 6.** Additionally, the knowledge score showed a negative correlation with stigma.

**Table 6:** Degree of correlation between knowledge, attitude, practices and stigma score.

Score	N	Correlation co-efficient	P-value
Knowledge-attitude score	547	0.32	<0.01
Attitude-practice score	619	0.11	0.01
Knowledge-practice score	594	0.20	<0.01
Knowledge- stigma score	553	-0.27	<0.01

### Determinants of knowledge, attitude and practice scores

In regression analysis for knowledge and attitude scores, the models for knowledge and attitude were significant with an R^2^ value of 0.027, 0.152 respectively **(S1 Table)**. Age (B = 0.36, C.I = 0.11 to 0.62, p-value<0.01) and main source of information (B = 0.83, C.I 0.38–2.20, p-value<0.05) were the only significant determinants of the knowledge score. For the attitude score, education (B = 0.25, C.I = 0.25 to 0.35, p-value<0.01) and knowledge scores (B = 0.21, C.I = 0.15 to 0.26, p-value<0.01) were significant determinants.

## Discussion

The mean knowledge, attitude and practice scores for participants were 4.06 (SD = 1.67, range 0–8), 2.46 (SD = 1.18, range 0–4), and 3.65 (SD = 0.73, range 0–4), respectively. Factors associated with the knowledge, attitude and practice scores were education, occupation, age and primary source of information. There was a positive correlation between knowledge and attitude, whereas there was a negative correlation seen between knowledge and stigma. On multivariable regression analysis, age and primary source of information was a significant determinant of knowledge score while education and knowledge score were significant determinants of attitude score.

The COVID-19 pandemic continues to spread and has posed as a major public health challenge at global, national and local levels. Although the vaccination drive is ongoing [35], it is important to ensure that people continue to adopt the preventive and promotive behaviours such as washing hands regularly, social distancing and wearing a mask [36]. For promoting preventative practices and attitude, knowledge about disease and expected behaviour is very important [37].

In the present study, knowledge among the study participants was found to be poor with a mean score of 4.06 and 50.7% correct answer rates. Score of 80% and above determines sufficient knowledge, a cut-off identified by Olum et al. [38]. This finding is in contrast with other studies done in other parts of India, Asia and similar LMICs that have shown satisfactory or good levels of knowledge. This difference can be attributed to the fact that our study caters to the poor and marginalized communities of central-India with low access to internet, as compared to other studies which were primarily done among internet users of more accessible areas. In our study, a majority of people reported their main source of information to be radio, TV and newspaper while the proportion of people relying on internet, friends or relatives and local health facility was comparatively smaller. It was also seen that the mean knowledge score was higher for those who relied on radio, TV and newspaper than those who identified no primary source of information. This highlights the need to promote and make accessible alternate information sources through these media platforms. These findings are in accordance with a multi-national study done by John Hopkins Center for Communication Programs that states that the exposure to information from radio, TV, Newspaper and Internet is not only higher in India but is also associated with more trust on the information source [39]. Therefore, it is vital to tap into the potential of these communication platforms to disseminate the right information in a timely manner.

Among the 8 questions, knowledge about common symptoms of COVID-19 infection and washing hand frequently was relatively low at 35.3% and 40.3%, respectively. The results suggest strengthening of preventive approach through feasible and effective risk communication strategies. It is also a matter of great concern that 53% of participants did not know that COVID-19 infection can be spread by asymptomatic person. This lack of awareness may negatively influence the COVID-19 appropriate behaviours. More than three-fourth of study participants were aware of the fact that people with co-morbidities such as diabetes, heart disease, and obesity are at higher risk which is similar to the findings of a study conducted in Maharashtra by Shukla S et al. [40].

Our study revealed that almost one-third of the participants reported worrying all the time about contracting COVID-19. According to a global survey done by YouGov [41], Indians are more fearful of COVID-19 compared to western countries but are less scared than other Asian economies. The finding of our study is similar to a study conducted by Grover et al. which found that more than one-third respondents reported fear and anxiety of acquiring COVID-19 infection [42]. This might be because the survey was conducted after lock-down and people might have witnessed impact of lockdown on household income, and social lifestyle or because of mysterious nature of the virus. Often fear results into stigma and discrimination. A section of society is at higher risk of COVID-19, so the issue of stigma needs special emphasis.

When asked if patients who have recovered from COVID-19 should be allowed to enter their locality and if health care workers treating COVID-19 should be allowed to enter their locality, over a third of the respondents replied in the negative. This indicates a high prevalence of stigma among the participants. This is similar to another study done among housekeeping and janitors where a similar apprehension was seen in inclusion of COVID-19 recovered patients in mainstream society [43] This study found negative correlation between knowledge and stigma, which suggests that correct information is crucial for addressing stigma. This is important from the public health standpoint, because in the past, we have witnessed fear and stigma undermining the public health efforts towards control and elimination of various diseases such as HIV/AIDS, Tuberculosis etc. [44] It also has serious mental health implications on the recovered patients and health care workers [45]. In a country like India, where violence against healthcare workers was already prevalent and on the rise, the stigma further fueled violence against doctors and health workers with many reports of discrimination and attacks on health workers by patient attendants, communities and landlords [46]. Our study suggests that over 80% participants reported always wearing a mask whenever they went outside in the past few days and washing hands with soap and water frequently. Around 15% of the participants reported not following the practice of wearing mask and frequent hand washing regularly. The findings are in contrast with the John Hopkins survey where the practice was lower(60–70%) in rural areas [39]. It should be noted that the studies have mostly reported self-reported practices and may not be reflective of the actual picture.

The KAP scores are significantly higher in those with higher education, those who were salaried employees over the agricultural/daily wage labours and farmers. Also, the middle-aged (36–55 yrs) and those between 16-35yrs fared better in practice than younger respondents less than 16 years. There was no difference between the KAP scores of males and females. These findings emphasize on the need to target risk communication messages to the vulnerable population and those with lack of access to reliable knowledge such as those with lower education, farmers, agricultural/daily age laborers. Our study found moderate correlation between knowledge and attitude, suggesting that higher knowledge will help in inculcating positive desirable attitudes towards COVID-19. This is in line with the findings from other studies [47, 48].

Our study findings are in contrast to other studies done using online survey methods, where a higher KAP score was depicted [49, 50]. This could be attributed to the difference in the survey platforms and the communities involved. Online platforms have reflected higher KAP due to higher reach and therefore, higher awareness regarding COVID-19. This further reinforces the need to reach out to the populations with low internet usage through alternate channels. However, the practices reported were similar in the present study and these studies, possibly because being self-reported information, respondents reported a higher level of adherence to COVID-19 appropriate behaviours.

Our study found age and source of information to be the main determinants of knowledge. Whereas education and knowledge scores were the significant determinants for higher attitude scores. These determinants are similar to findings from other studies which have also stated education, knowledge and source of information to be important determinants [5].

### Strengths and limitations

To the best of our knowledge, this is the first survey assessing the knowledge, attitude and practices related to COVID 19 among poor and marginalized populations of the states of Madhya Pradesh and Jharkhand, who do not have a high degree of internet penetration. The usage of the Interactive Voice Response system for data collection facilitated inclusion of a large number of respondents, which would not have been possible in a short time through house-to-house survey, considering the restrictions which were in place at the time of survey. The use of IVRS platform also facilitated reaching out to a category of participants who are usually missed out in internet-based surveys or studies–a lot of which were conducted to answer similar research questions during the same period. The insights from this baseline assessment are being used to design effective risk communication messages and information regarding COVID-19.

There are some limitations of the study. Due to limitations of the usage of IVRS platform, there were missed responses for the questions. Secondly since the survey participation was respondent-driven, there may have been a selection bias and hence findings may not be completely representative of the study population. Also, since it was a self-reported survey, there is a possibility that the responses towards attitude and practices may be influenced by social desirability bias and is not reflective of the actual scenario.

### Policy recommendations

Extensive risk communication needs to be done with relevant and updated information through alternate media channels such as local media platforms, radio and newspapers which are accessed by the marginalized communities where internet penetration is not high.

Targeted interventions need to be made for knowledge dissemination regarding COVID-19 among the vulnerable population as these populations are more at risk and less likely to be able to follow social distancing measures.

Dispelling myths and tackling misinformation is important as correct information is directly correlated with more adherence to COVID-appropriate behaviour and lesser stigma.

## Conclusion

The knowledge, attitude and practices related to COVID-19 are low among the poor and marginalized communities of central India. There is high prevalence of stigma in these communities. Knowledge is positively correlated with attitude and practices whereas it is negatively correlated with stigma. Therefore, it is necessary to provide effective risk communication messages to these communities through alternate channels.

## Supporting information

S1 Appendix Survey questionnaire. (DOCX)

S1 Table Linear regression of knowledge and attitude scores. (DOCX)

S1 File Dataset. (XLSX)

## Abbreviations

SD: Standard Deviation
LMICs: Low-and-middle income countries
SARS: Severe Acute Respiratory Syndrome
WHO: World Health Organization
KAP: Knowledge, Attitude and Practices
C.I: Confidence Interval
USAID: The United States Agency for International Development
IVRS: Interactive Voice Response System
ICMR: Indian Council of Medical Research
IRB: Institutional Review Board
