# Supplementary material for: Knowledge, attitude, and practices related to COVID-19 among poor and marginalized communities in central India: A cross-sectional study
Source: PLoS One. 2022 Apr 6;17(4):e0264639. doi: 10.1371/journal.pone.0264639 (PMC8986002; doi:10.1371/journal.pone.0264639)
Supplement: S1 Appendix — (DOCX) [file pone.0264639.s001.docx]

**ANNEXURE**

**KAP QUESTIONNAIRE**

**Socio-Demographic Parameters**

**1.District:**

a. Barmani b. Guna c. Khandwa d. Rajagarh e. Ranchi

**2. Age:**

a. Less than 16yrs b. 16 to 35 yrs c. 36 to 55 yrs d. More than 55 yrs

**3. Gender**

a. Male b. Female c. Others

**4. Occupation**

a. Agriculture or daily wage labor b. Farmer

c. housewife or house husband d. salaried employee

e. student f. unemployed

g. self employed

**5. Marital Status**

a. Unmarried b. Married c. Divorced/Widowed

**6. Education:**

a. No formal education b. Up to 5

c. 6-12 d. Diploma graduation Post graduation

**Short Survey:**

**1. Which of the following are common symptoms of CORONA infection?**

(a) Loss of appetite and weight loss (b) Itching of skin and rashes

(c) Fever and dry cough (d) Don't know

**2. By which of the following ways does CORONA infection spread?**

(a) Drinking contaminated water (b) Respiratory droplets from infected person

(c) Mosquito bites (d) Don't know

**3. Which of the following steps can one take to prevent CORONA infection?**

(a) Drinking hot/warm water frequently

(b) Washing hands with soap and water frequently

(c) Standing in sun daily at least for 30 minutes

(d) All of the above

(e) Don't know

**4. What should one do if someone has symptoms of CORONA infection?**

(a) Call the CORONA helpline and follow the provided instructions

(b) Visit the nearest health facility

(c) Visit the nearest CORONA hospital

(d) Take home remedies and avoid going out

(e) Don't know

**5. Which among the following is your main source of information on CORONA infection?**

(a) Radio, TV or News paper

(b) Friends and relatives

(c) Internet

(d) Local health worker like Anganwadi, ANM or ASHA or Local health facility

(e) No information source

**6. Do you worry about contracting CORONA infection?**

(a) Yes, all the time (b) Yes, many times

(c) Yes, some times (d) No

**Long Survey**

**Section A**

1. **A minimum distance of how many meters from another person is necessary for protection against CORONA infection?**(a) 1 meter
   (b) 2 meters
   (c) 3 meters
   (d) Don't know

**Section B: Listen carefully to the following statements on CORONA infection and answer whether they are true or false.**

1. **People with CORONA infection who do not have any symptoms cannot spread infection to others.**(a) True
   (b) False
   (c) Not sure
2. **Children and teenagers do not need to make efforts to prevent CORONA infection because they have a strong immune system.**(a) True
   (b) False
   (c) Not sure
3. **People with CORONA infection who have chronic diseases such as diabetes, heart disease, and obesity are at higher risk.**(a) True
   (b) False
   (c) Not sure
4. **If a crowd happens due to religious purpose, there is no chance of CORONA infection spreading.**(a) True
   (b) False
   (c) Not sure

**Section C:**

1. **In the past few days, have you worn a mask when you were outside?**(a) Always
   (b) Occasionally
   (c) Never
2. **In the past few days, have you been washing your hands with soap and water frequently?**(a) Always
   (b) Occasionally
   (c) Never
3. **For the safety of people in your area, patients who have recovered from Corona infection should not be allowed to live in your area.**(a) Agree
   (b) Disagree
   (c) Not sure
4. **For the safety of people in your area, healthcare workers taking care of CORONA patients, should not be allowed to visit your area.**(a) Agree
   (b) Disagree
   (c) Not sure
