# Supplementary material for: Knowledge, attitude, and practices related to COVID-19 among poor and marginalized communities in central India: A cross-sectional study
Source: PLoS One. 2022 Apr 6;17(4):e0264639. doi: 10.1371/journal.pone.0264639 (PMC8986002; doi:10.1371/journal.pone.0264639)
Supplement: S1 Table — (DOCX) [file pone.0264639.s002.docx]

**Table S1: Linear regression of knowledge and attitude scores**

| **Dependent Variable** | **Parameter** | **Categories** | **B** | **95% Confidence Interval** | | **Significance** |
| --- | --- | --- | --- | --- | --- | --- |
|  |  |  |  | **L.L.** | **U.L** |  |
| **Knowledge score^*^** | **Intercept** | **-** | 2.61 | 0.94 | 4.28 | <0.01 |
|  | **Age** | **Less than 16yrs** | -0.92 | -2.27 | 0.41 | 0.17 |
|  |  | **16-35yrs** | -0.51 | -1.80 | 0.77 | 0.43 |
|  |  | **36-55 yrs** | -0.09 | -1.41 | 1.22 | 0.88 |
|  |  | **more than 55 yrs** | 0^a^ | . | . | . |
|  | **Gender** | **Female** | 1.09 | 0.11 | 2.06 | 0.08 |
|  |  | **Male** | 1.09 | 0.13 | 2.05 | 0.06 |
|  |  | **Others** | 0^a^ | . | . |  |
|  | **Marital Status** | **Divorced/Widowed** | 0.12 | -0.95 | 1.20 | 0.83 |
|  |  | **Married** | 0.19 | -0.15 | 0.55 | 0.27 |
|  |  | **Unmarried** | 0^a^ | . | . | . |
|  | **Occupation** | **Housewife or house-husband** | -0.40 | 0.34 | -1.25 | 0.34 |
|  |  | **Student** | -0.16 | 0.56 | -0.73 | 0.56 |
|  |  | **Self-employed** | -0.55 | 0.12 | -1.25 | 0.12 |
|  |  | **Salaried employee** | -0.31 | 0.33 | -0.94 | 0.33 |
|  |  | **Farmer** | -0.49 | 0.09 | -1.07 | 0.09 |
|  |  | **Agriculture or daily wage labour** | -0.26 | 0.37 | -0.85 | 0.37 |
|  |  | **unemployed** | 0^a^ | . | . | . |
|  | **Education** | **Diploma Graduation or Post graduation** | 0.32 | 0.19 | -0.16 | 0.19 |
|  |  | **6 to 12** | 0.18 | 0.38 | -0.23 | 0.38 |
|  |  | **up to 5** | 0.36 | 0.14 | -0.12 | 0.14 |
|  |  | **No formal education** | 0^a^ | . | . | . |
|  | **Main source of information** | **Friends and relatives** | 0.56 | 0.16 | -0.23 | 0.16 |
|  |  | **Local health facility/ worker t** | 0.88 | 0.03 | 0.07 | 0.03 |
|  |  | **Radio, TV, Newspaper** | 1.07 | 0.00 | 0.40 | <0.01 |
|  |  | **internet** | 0.80 | 0.03 | 0.05 | 0.03 |
|  |  | **No source** | 0 ^a^ | . | . |  |

| **Attitude score^#^** | **Intercept** | **-** | 1.59 | 0.44 | 2.73 | <0.01 |
| --- | --- | --- | --- | --- | --- | --- |
|  | **Age** | **Less than 16yrs** | -0.19 | -1.09 | 0.69 | 0.66 |
|  |  | **16-35yrs** | -0.06 | -0.92 | 0.78 | 0.87 |
|  |  | **36-55 yrs** | -0.01 | -0.88 | 0.86 | 0.97 |
|  |  | **more than 55 yrs** | 0 ^a^ | . | . | . |
|  | **Gender** | **Female** | -0.12 | -0.89 | 0.63 | 0.74 |
|  |  | **Male** | -0.23 | -0.98 | 0.52 | 0.54 |
|  |  | **Others** | 0 ^a^ | . | . | . |
|  | **Marital Status** | **Divorced/Widowed** | 0.19 | -0.53 | 0.93 | 0.60 |
|  |  | **Married** | -0.18 | -0.43 | 0.05 | 0.13 |
|  |  | **Unmarried** | 0 ^a^ | . | . | . |
|  | **Occupation** | **Housewife or house-husband** | -0.38 | -0.95 | 0.18 | 0.182 |
|  |  | **Student** | -0.00 | -0.39 | 0.37 | 0.96 |
|  |  | **Self-employed** | 0.13 | -0.34 | 0.62 | 0.57 |
|  |  | **Salaried employee** | 0.05 | -0.38 | 0.49 | 0.79 |
|  |  | **Farmer** | -0.04 | -0.43 | 0.34 | 0.81 |
|  |  | **Agriculture or daily wage labour** | 0.01 | -0.39 | 0.41 | 0.95 |
|  |  | **unemployed** | 0 ^a^ | . | . | . |
|  | **Education** | **Diploma Graduation or Post graduation** | 0.77 | 0.42 | 1.11 | <0.01 |
|  |  | **6 to 12** | 0.33 | 0.03 | 0.62 | 0.02 |
|  |  | **up to 5** | 0.13 | -0.21 | 0.47 | 0.45 |
|  |  | **No formal education** | 0 ^a^ | . | . | . |
|  | **Main source of information** | **Friends and relatives** | 0.41 | -0.13 | 0.96 | 0.14 |
|  |  | **Local health facility/ worker t** | -0.04 | -0.61 | 0.52 | 0.87 |
|  |  | **Radio, TV, Newspaper** | 0.10 | -0.34 | 0.55 | 0.65 |
|  |  | **internet** | -0.04 | -0.55 | 0.46 | 0.85 |
|  |  | **No source** | 0 ^a^ | . | . | . |
|  | **knowledge score** |  | 1.59 | 0.15 | 0.27 | <0.01 |
| ^a = Reference group^  *Knowledge score diagnostics:  There was linearity as assessed by partial regression plots and a plot of studentized residuals against the predicted values. There was independence of residuals, as assessed by a Durbin-Watson statistic of 1.8. There was homoscedasticity, as assessed by visual inspection of a plot of studentized residuals versus unstandardized predicted values. There was no evidence of multicollinearity, as assessed by Pearson correlation co-efficient more than 0.7 between the variables and Tolerance value >0.1. There were no studentized deleted residuals greater than ±3 standard deviations. The assumption of normality was met, as assessed by a Q-Q Plot. The multiple regression model statistically significantly predicted knowledge score, F(6, 570) = 2.597, p =0.017 , . R2 = .027.  #Attitude score diagnostics:  There was linearity as assessed by partial regression plots and a plot of studentized residuals against the predicted values. There was independence of residuals, as assessed by a Durbin-Watson statistic of 1.74. There was homoscedasticity, as assessed by visual inspection of a plot of studentized residuals versus unstandardized predicted values. There was no evidence of multicollinearity, as assessed by Pearson correlation co-efficient more than 0.7 between the variables and Tolerance value >0.1. There were no studentized deleted residuals greater than ±3 standard deviations. The assumption of normality was met, as assessed by a Q-Q Plot. The multiple regression model statistically significantly predicted knowledge score, F(7, 505) = 14.11, p <0.01 , R2 = .152 | | | | | | |
